# Validation of an interactive electronic book for cardiovascular risk reduction in people living with HIV^*^

**DOI:** 10.1590/1518-8345.5568.3512

**Published:** 2022-03-11

**Authors:** Elizabete Santos Melo, Marcela Antonini, Christefany Régia Braz Costa, Priscila Silva Pontes, Elucir Gir, Renata Karina Reis

**Affiliations:** 1 Universidade Paulista, Instituto de Ciências da Saúde, São José do Rio Preto, SP, Brasil.; 2 Universidade Brasil, Campus Fernandópolis, Fernandópolis, SP, Brasil.; 3 Bolsista da Coordenação de Aperfeiçoamento de Pessoal de Nível Superior (CAPES), Brasil.; 4 Universidade de São Paulo, Escola de Enfermagem de Ribeirão Preto, Centro Colaborador da OPAS/OMS para o Desenvolvimento da Pesquisa em Enfermagem, Ribeirão Preto, SP, Brasil.; 5 Bolsista do Conselho Nacional de Desenvolvimento Científico e Tecnológico (CNPq), Brasil.

**Keywords:** HIV, Cardiovascular Diseases, Health Education, Health Literacy, Educational Technology, Validation Study, HIV, Doenças Cardiovasculares, Educação em Saúde, Letramento em Saúde, Tecnologia Educacional, Estudo de Validação, VIH, Enfermedades Cardiovasculares, Educación en Salud, Alfabetizacíon em Salud, Tecnología Educacional, Estudio de Validación

## Abstract

**Objective:**

To validate interactive digital educational material in the form of an electronic book about the prevention and reduction of cardiovascular risk from the perspective of people living with the Human Immunodeficiency Virus.

**Method:**

This is a methodological study based on the theory of evaluation research, of the results analysis type, which involves technological production. The data were collected throughout Brazil by means of a virtual questionnaire consisting of items for general, visual, language, usability, content and appearance assessment of the educational material. A minimum Agreement Index of 80% was adopted in order to certify validity.

**Results:**

309 individuals living with the Human Immunodeficiency Virus participated in the study, the majority (84.3%) being male, aged between 19 and 65 years old and with complete higher education (29.3%). More than 90% of the participants assessed the book as suitable for solving doubts and providing preventive care for cardiovascular health. All the items evaluated reached an index above 0.80. The general evaluation of all the items reached a mean of 0.92, with general assessment (0.97) and content assessment (0.94).

**Conclusion:**

The educational material proved to be valid, adequate and relevant to promote literacy in health, and could contribute to health promotion and to the prevention of cardiovascular diseases.

## Resumo

**Objetivo:**

Validar um material educativo digital interativo no formato de livro eletrônico sobre prevenção e redução do risco cardiovascular na perspectiva das pessoas vivendo com vírus da imunodeficiência humana.

**Método:**

Trata-se de um estudo metodológico baseado na teoria de pesquisa de avaliação, do tipo análise de resultados, que envolve produção tecnológica. Os dados foram coletados em todo o Brasil por meio de um questionário virtual composto por itens para avaliação geral, visual, linguagem, usabilidade, conteúdo e aparência do material educativo. Para atestar a validade, adotou-se o Índice de Concordância mínimo de 80%.

**Resultados:**

Participaram do estudo 309 pessoas vivendo com vírus da imunodeficiência humana, a maioria (84,3%) do sexo masculino, com idade entre 19 e 65 anos e ensino superior completo (29,3%). Mais de 90% dos participantes avaliaram o livro como adequado para tirar dúvidas e realizar cuidados preventivos à saúde cardiovascular. Todos os itens avaliados alcançaram índice acima de 0,80. A avaliação geral de todos os itens alcançou média de 0,92, sendo a avaliação geral (0,97) e o conteúdo (0,94).

**Conclusão:**

O material educativo mostrou-se válido, adequado e pertinente para promover a alfabetização em saúde, e poderá contribuir com a promoção da saúde e prevenção de doenças cardiovasculares.

## Resumen

**Objetivo:**

Validar un material educativo digital interactivo en forma de libro electrónico sobre prevención y reducción del riesgo cardiovascular desde la perspectiva de las personas que viven con el virus de la inmunodeficiencia humana.

**Método:**

Se trata de un estudio metodológico basado en la teoría de la investigación evaluativa, del tipo análisis de resultados, que incluye producción tecnológica. Los datos fueron recolectados en todo Brasil a través de un cuestionario virtual compuesto por ítems de evaluación general e ítems que evalúan el aspecto visual, el lenguaje, la usabilidad, el contenido y la apariencia del material educativo. Para comprobar la validez se adoptó un Índice de Concordancia mínimo del 80%.

**Resultados:**

Participaron en el estudio 309 personas que viven con el virus de la inmunodeficiencia humana, la mayoría (84,3%) era del sexo masculino, tenía entre 19 y 65 años y estudios superiores completos (29,3%). Más del 90% de los participantes calificaron el libro como adecuado para aclarar dudas y realizar cuidados preventivos para la salud cardiovascular. Todos los ítems evaluados alcanzaron un índice superior a 0,80. La evaluación general de todos los ítems alcanzó una media de 0,92, la evaluación general fue 0,97 y la de contenido 0,94.

**Conclusión:**

El material educativo demostró ser válido, adecuado y relevante para promover la alfabetización en salud y puede contribuir a la promoción de la salud y a la prevención de enfermedades cardiovasculares.

**Highlights**

(1) More than 80% of the participants reported motivation to read the e-book. (2) The e-book is relevant for planning cardiovascular health self-care. (3) It is a safe and reliant material as a facilitator for a lifestyle change. (4) Health literacy of PLHIV through digital technologies such as e-books. (5) The e-book is available for free download and can reach PLHIV.

## Introduction

The advent and large-scale availability of the anti-retroviral therapy (ART) has transformed the Human Immunodeficiency Virus (HIV) infection into a chronic disease^1^. Treatment reduced the number of deaths attributed to opportunistic infections and increased the longevity of people living with HIV (PLHIV). However, other chronic comorbidities have emerged, such as cardiovascular diseases (CVDs), which became one of the main causes of morbidity in this population^2^ ^-^ ^3^.

Traditionally, CVDs result from the interaction of multiple risk factors dichotomized between modifiable (smoking, inadequate diet, sedentary lifestyle, dyslipidemia, hypertension and diabetes) and non-modifiable (advanced age and family history)^4^ ^-^ ^5^. CVDs are also related to long-term persistent metabolic disorders, such as insulin resistance and altered fat distribution (lipodystrophy)^4^ ^-^ ^6^.

In addition, a number of studies have shown that these risk factors for CVDs are more frequent in PLHIV undergoing regular ART, evidencing living with the infection as a specific risk factor for CVDs^3^ ^,^ ^6^ and that, added to the traditional factors, they predispose PLHIV to an even greater risk for the development of diseases^6^ ^-^ ^7^. 

Thus, changing the lifestyle habits, such as increased physical activity, weight reduction and education about healthy eating practices is essential to mitigate the modifiable risk factors for CVDs in PLHIV^7^ ^-^ ^8^. Such changes can be achieved through education in health, which seeks to increase people’s empowerment in relation to their health care^9^, as well as improving literacy in health, which plays a prominent role in the primary and secondary prevention of CVDs^10^. 

Literacy in health has emerged as a major area of interest, being essential for research and health care in various chronic disease conditions. It is defined as “the degree to which individuals can find, understand and use information and services to make health-related decisions and initiate actions for themselves and others”^11^. Low literacy in health is associated with challenges for those living with HIV, including drug non-adherence and worse health outcomes^12^.

Difficulty accessing reliable information and/or lack of understanding are significant barriers to the effectiveness of literacy in health^13^ ^-^ ^14^; therefore, the elaboration of educational materials in accessible language, as well as their validation by their target audience, can contribute to reducing such barriers.

In this context, digital educational materials, including the interactive electronic book (e-book), are information technologies that have provided a greater degree of interaction with health knowledge^15^, especially when compared to passive learning carried out through static information sources^16^. 

E-books represent an important strategy to improve literacy in health and work on self-care through behavioral change, complementing the health team’s traditional educational efforts to reduce risk and prevent CVDs among PLHIV^16^. 

Therefore, this study aimed at validating interactive digital educational material in the form of an e-book about the prevention and reduction of cardiovascular risk from the perspective of people living with HIV/AIDS (PLHIV). 

## Method

### Study design

This study was part of a larger project entitled “Development, validation and effectiveness of educational technologies focused on the behavior, preventive practices and lifestyle of people living with HIV/AIDS”. This was a methodological study based on the theory of evaluation research, of the results analysis type, which involves technological production^17^. The e-book focused on reducing cardiovascular risk in PLHIV. 

The study was conducted in three stages. The first two began with the process of construction and validation of content and appearance by health experts (in relation to content and layout) and with experts in the field of information technology (evaluating the interfaces and functionalities of the e-book). For this, a literature review was conducted and then the book was built in digital format and validated, as described in the previous study^18^.

In this third stage, in order to continue the validation process of the material, validation was carried out with the target audience regarding the general assessment and aspects such as visual, appearance, language, usability and content of the e-book in order to obtain the final version of the material^19^. 

The e-book entitled “Take care of your heart: Strategies to reduce cardiovascular risk in people living with HIV/AIDS”^20^ has its interface built in HTML5 and Java Script and generated a file in the Electronic Publication (ePUB) format. The content was written in order to meet the needs of the population; therefore, an accessible language with no technical terms was used to assist in understanding the content. Thus, in order to facilitate its interpretation, infographics and videos were created to make the Virtual Learning Environment (VLE) more dynamic and interactive^18^. 

The e-book was built with the aim of promoting usability and accessibility^18^, providing the user with an easy-to-use, dynamic and interactive tool, and is available for free download at Apple Books on the iOS® platform or in Google Play on the Android® platform, being able to broadly reach PLHIV throughout Brazil. 

In general, the material includes guidelines on knowledge about the risk for CVDs and addresses aspects of the modifiable risk factors: smoking, sedentary lifestyle, stress, obesity, diabetes mellitus, hypertension and dyslipidemia. In addition, the content about the risk for CVDs in PLHIV and the interventions/strategies for their reduction was assessed, including healthy eating, smoking cessation, encouragement of physical activity and stress relief based on the scientific literature^4^ ^,^ ^19^ ^,^ ^21^. 

### Study locus

Data collection was carried out in a virtual environment with national coverage in the five Brazilian regions (North, Northeast, Midwest, Southeast and South).

### Period

Data collection covered the period from March 31^st^, 2020 to March 1^st^, 2021.

### Population, selection criteria and sample

The study sample consisted of 312 people living with HIV who were literate and had access to the Internet. The following inclusion criteria were established: knowledge of one’s HIV status, regardless of the stage of infection, being over 18 years old, literate and having access to the Internet. Three participants who did not complete the questionnaire were excluded. Thus, the sample consisted of 309 people living with HIV who met the selection criteria.

### Study variables

The *online* quiz was elaborated containing sociodemographic and behavioral variables: gender, age (full years old), schooling, current work situation, individual income (minimum wages), Brazilian region of residence.

### Instrument used to collect the information

For validation with the participants belonging to the target audience, a questionnaire was developed that assessed the visual aspect, language, usability, content, appearance and general evaluation of the educational material, on a Likert-type scale, with five levels of judgment^22^. For the general evaluation: Excellent, Good, Indifferent, Bad and Very Bad; for the visual variable: Excellent, Good, Fair, Bad and Very Bad; for the language, usability, content and appearance variables: Totally Agree (TA), Agree (A), Neither Agree nor Disagree (N), Totally Disagree (TD) and Disagree (D). 

### Data collection

To carry out data collection, invitations were sent to PLHIV groups throughout Brazil via social media (Facebook® and Instagram®) and in WhatsApp® groups, as well as on the profile of the research group on the pages of these social media. In addition, the invitation was also posted on official pages of research partners and other researchers and health professionals from different regions of the country. Fliers with a QR code were also printed for direct access to the study questionnaire and distributed in Specialized Care Services (SCSs) to PLHIV.

The recruitment invitation consisted of a brief exposition text presenting the study with the inclusion criteria followed by the link or QR code that directed the participants to the virtual data collection platform (Survey Monkey®). The study home page already presented the full Free and Informed Consent Form (FICF). After reading it, the participants were able to select two possible answer options: “I agree to participate in the research” or “I do not agree to participate in the research”. Those who agreed to participate in the study received the online questionnaire, which was made available for access via computers or mobile devices (cell phones). 

### Data analysis

The data collected on the Survey Monkey® platform were transferred to an Excel spreadsheet and to the Statistical Package for the Social Sciences (SPSS)® statistical program, version 22.0. Descriptive statistical analyses were performed by calculating absolute and relative frequencies for the qualitative variables and, for the quantitative variables, calculations of central tendency (mean) were used. To certify the validity of each item addressed in the assessment instruments, a minimum Agreement Index (CI) of 80% was adopted among the participants, following the reference values of other validation studies^23^ ^-^ ^24^.

To assess the degree of inter-rater agreement, the inter-rater reliability coefficient was used (first-order agreement coefficient - AC1). AC1 has the advantages of resistance in relation to marginal homogeneity and the prevalence trait, in addition to having the same interpretation of the Kappa statistic [slight (0.0-0.2); acceptable (0.21-0.40); moderate (0.41-0.60); considerable (0.61-0.80); and almost perfect (0.81-1.00)]^25^.

In addition to that, the variables were classified as proposed in 1997^25^, separating the questions into Group 1: language, usability, content, appearance and Group 2: pictures, motivation to read, topics covered and indication of the e-book to someone else. 

### Ethical aspects

The project was submitted and approved by the Research Ethics Committee, under opinion number 3,915,295. Data confidentiality and anonymity was guaranteed to all the research participants.

## Results

The 309 PLHIV who participated in the study came from 25 states, covering the five Brazilian regions, with the largest share being from the Southeast with 50.5% (n=156) and the Northeast with 24.3% (n=75). Table 1 shows that the sample was predominantly made up of men, with 84.8% (n=262), aged between 19 and 65 years old and with a mean of 32.7 years old (±9.6). 

In addition, most of the participants had completed higher education, 29.1% (n=90); 65.0% (n=201) were inserted in the formal or informal labor market; and 38.2% (n=118) had an income of one to two minimum wages.

**Table 1:** Characterization of the people living with HIV/AIDS regarding the sociodemographic variables (n=309). Brazil, 2020-2021

Variables	n (309)	% (100)
**Gender**		
Male	262	84.8
Female	47	15.2
**Schooling**		
Incomplete Elementary School	06	1.9
Complete Elementary School	07	2.2
Incomplete High School	09	2.9
Complete High School	44	14.2
Incomplete Higher Education	81	26.2
Complete Higher Education	90	29.1
Graduate studies	72	23.3
**Work Situation**		
Active	201	65.0
Inactive	87	28.1
Retired	05	1.6
Distanced from work	16	5.2
**Region**		
North	14	4.5
Northeast	75	24.3
Midwest	22	7.1
Southeast	156	50.5
South	42	13.6
Individual income (minimum wages)*		
No income	24	7.8
<1	54	17.5
1-2	118	38.2
3-4	45	14.6
More than 4	68	22.0

*Minimum Wage 2020 (Brazil): R$ 1,045.00 (one thousand and forty-five reais)

Table 2 presents the answers and the Agreement Index (AI) of each assessment item among them. In the general evaluation of all the items, a mean of 0.92 was reached in the AI. The general evaluation and content items obtained the highest AIs with values equal to 0.97 and 0.94, respectively; and the item about appearance had the lowest value (0.86) in the AI. All the other items evaluated reached an AI above 0.80.

**Table 2:** Assessment of the agreement among the participants regarding adequacy and relevance of the e-book (n=309). Brazil, 2020-2021

Evaluation items						
**General Evaluation**	Excellent	Good	Indifferent	Bad	Very bad	AI
In general, how do you assess the e-book	160	141	08	00	00	0.97
Visual	Excellent	Good	Fair	Bad	Very bad	AI
What did you think about the e-book’s visual aspect?	145	141	00	21	02	0.93
Language	TA*	A^†^	N^‡^	D^§^	TD^║^	AI^¶^
Is the language used in the information easy to understand?	130	145	32	02	00	0.89
**Usability**						
Is the e-book easy to use?	128	156	24	01	00	0.92
**Content**						
Is the content covered relevant to your health?	161	130	18	00	00	0.94
Can the guidelines contained in the e-book help you improve your health?	152	138	19	00	00	0.94
**Appearance**						
Are the e-book’s videos interesting?	116	150	43	00	00	0.86

*TA = Totally agree; ^†^A = Agree; ^‡^N = Neither agree nor disagree; ^§^D = Disagree; ^||^TD = Totally disagree; ^¶^AI = Agreement Index

When asked, most of the 293 participants (94.8%) indicated that educational materials can help solve doubts about the prevention of cardiovascular diseases. Of the total, 301 (97.4%) highlighted that the issues addressed are necessary for the patients with HIV to be able to perform the adequate care measures for cardiovascular health.

**Figure 1:**
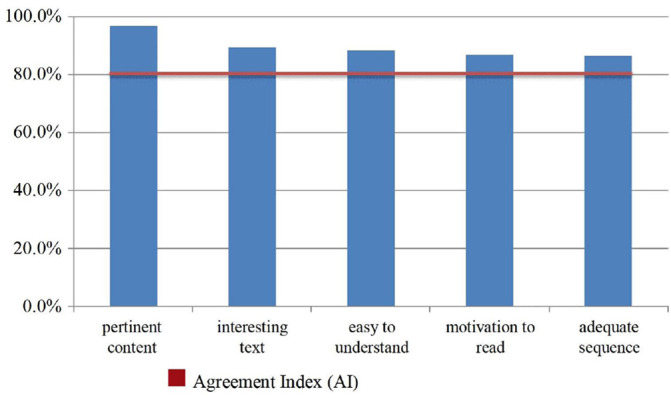
Participants’ agreement regarding pertinence of the content, understanding, interest and motivation for reading (n=309). Brazil, 2020-2021

When asked about pertinence of the content, understanding, motivation for reading and sequencing of the e-book, the participants indicated approval with more than 80% of agreement in all questions, with emphasis on the contents addressed, pointed out as pertinent by 97.4% (n=301) of the participants (Figure 1). 

Table 3 shows the data related to the assessment of the inter-rater agreement of the e-book, which showed “almost perfect agreement” among the evaluators regarding the aspects assessed in Group 2 and “acceptable agreement” among the evaluators regarding the aspects assessed in Group 1.

**Table 3:** Inter-rater agreement measure related to the validation of the e-book as for adequacy and pertinence (n=309). Brazil, 2020-2021

Validation	*AC1	SE_AC1^†^	p-value
Group 1	0.287	0.017	0.0001
Group 2	0.921	0.061	0.0001

*AC1 = Inter-evaluator reliability coefficient; ^†^SE_AC1 = Standard Error of reliability among the evaluators. Note: Significance level: 5% (p<0.05).

## Discussion

In this study, it was verified that the *e-book* elaborated was considered valid from the perspective of the target audience in terms of visual, language, usability, content, appearance and general evaluation. It is noteworthy that validation of materials is an important stage for the development of educational materials^26^ and validation by its target audience allows identifying and working on the aspects that evidence its readability.

A study conducted in the United States showed that all types of educational materials related to cardiovascular health exceed the reading ability of their target audience and that this distance between readability of the material and the population’s reading level negatively affects understanding of the information offered and, consequently, success of their use as a basis for decision-making focused on self-care^13^.

Transposing this issue to the Brazilian reality, where the majority of the territorial population (50.4%) has not completed elementary school^27^, it is necessary to develop interactive educational materials, easy to understand and that promote and facilitate self-management in health for this population^14^.

The contents covered were considered pertinent and include aspects related to the main cardiovascular risk factors and guidelines aimed at prevention^4^ ^,^ ^28^. Our findings corroborate another Brazilian study, which points out that addressing CVDs among PLHIV is a priority and emphasizes the importance of managing both traditional and specific risk factors related to HIV infection to reduce the risk of CVDs in these individuals^29^. 

The construction and validation of interactive digital educational materials allows the general population to have access to relevant and appropriate contents for health promotion and disease prevention based on scientific evidence. This is relevant, since a large part of the content produced by science ends up without dissemination and/or practical application, feeding a vicious circle of exclusion of the majority of the population that does not have access to this means^30^.

Although CVDs have complex mechanisms and etiologies that are difficult to understand^13^, it was observed that, regarding readability of the e-book, the text was considered interesting, with an adequate sequence and easy to understand. 

The readability assessment is paramount and is considered as a quality metric that should be evaluated before online publication of any educational material^13^. This aspect is relevant, as educational materials and instructions routinely include technical language and complex explanations or do not contain understandable illustrations, making them difficult for the target audience to understand^10^. Clear and simple communication helps people feel more involved in their health care and increases their chances of following their care plans and adherence to the treatment^26^ ^,^ ^31^. 

In this context, the content and language addressed should also be highlighted, as they must be accessible and free of technical terms to assist in understanding the content, in addition to providing interactivity between the reader and the educational material. In addition to that, in the development of educational materials, the language must be objective, avoiding the use of long and detailed sentences, as they can provide dispersion and make reading tiring^31^.

In this study, more than 80.0% of the participants reported motivation to read the e-book and assessed the content addressed as pertinent to planning self-care for cardiovascular health. For the educational material to be current and relevant, it is indispensable that it is inserted in the social context of the target population, and that it meets its needs and particularities^32^ ^-^ ^33^. Therefore, themes and topics covered in educational contents must meet the needs of people, providing empowerment on the topic addressed^31^.

Regarding usability of the e-book, the study participants rated it as easy to use. It consists of videos, audios and links to access other pages that supplement the content addressed and make its use more playful, in addition to easy access and sharing through the social networks. Therefore, the ease of use of a tool is directly related to the level of satisfaction of the user who consumes it, since poorly designed interfaces can result in disinterest or discredit by the user^34^ ^-^ ^35^.

Educational materials made available virtually have been used as tools to improve knowledge, adherence to the treatment and self-care, from a perspective of health promotion and disease prevention^10^ ^,^ ^36^. When produced by health professionals, they have the power to strengthen the guidelines verbalized during the appointments, in addition to answering some questions. The approach used in the construction of the educational material allows identifying the needs of the target audience, especially when validation is carried out by such population.

Given the above, another challenging point is the basic use of digital resources (digital literacy) and the target audience’s ability to explore resources more broadly (digital literacy)^13^. In this study, the educational material was validated by PLHIV from all the Brazilian regions with the most varied types of schooling, although most had higher education and many attended graduate courses; therefore, they are consistent with the profile of individuals who have greater access to the Internet, information and more digital literacy. 

Thus, improving literacy in health among those with lower schooling levels represents a unique challenge for health professionals since, despite the benefits of using health information technology, it is possible that such information does not reach groups that perhaps have less access to the Internet and is included in groups aimed at this population on the social networks. Therefore, we suggest that this educational material is an educational resource option in the clinical practice for PLHIV.

A relevant aspect of this study is that it was validated by participants from all regions of the country, predominantly the Southeast and Northeast. The heterogeneity of Brazilian regions is related to the diversity of the HIV infection epidemiological profile in the country and to the different conditions of access to technologies across the states. 

In the historical evolution of AIDS in Brazil, the first cases were diagnosed in the Southeast until reaching the North and Northeast regions of the country. The proportional distribution of AIDS cases identified from 1980 to June 2019 shows different concentrations in the following Brazilian regions: Southeast (51.3%), South (19.9%), Northeast (16.1%), North (6.6%) and Midwest (6.1%)^37^. This heterogeneity of regions also implies peculiarities regarding the means of production, culture, education and access to the services, which makes health care not universal and equitable in Brazil, hindering access to diagnosis and treatment. The Southeast and South regions stand out in terms of the development of education, which exerts an impact on the detection, treatment and prevention of the disease. The Northeast region, on the other hand, is the second most populous region in Brazil and has one of the lowest indicators in the evaluation of the educational system’s performance^38^. 

Thus, our results reassert how the use of educational materials in digital format and the Internet show themselves as new possibilities that can help promote literacy in health, and even continuous and comprehensive care, transforming this technology into a new option for thinking and planning health interventions in the most varied scenarios.

In addition to knowledge, the subjects’ self-efficacy and motivation are considered as the basis for behavioral change^18^. Therefore, the use of technology, associated with quality information, can promote greater interest on the part of the subjects and, therefore, foster knowledge and motivation for change with a view to a better quality of life^18^.

In addition, we emphasize that the educational material can be used during Nursing consultations, as well as by other health professionals and/or literacy in health activities, to complement and reinforce the information provided. It is noteworthy that nurses play a fundamental role in the development of educational interventions in the health services and, in particular, they can benefit from the use of digital technologies to improve PLHIV’s literacy in health. 

Thus, we ratify the importance of elaborating and validating educational materials with the target population, in order to provide a safe and effective material that works as a facilitator for lifestyle change, seeking to encourage the performance of activities that promote healthier lifestyle habits. An easy-to-understand, interactive, accessible, usable, up-to-date and freely available content e-book is made available to PLHIV.

The limitation found in the study refers to the restriction of the results to groups that have access to the Internet, with a high schooling level and more used to handling computerized tools; therefore, with more ease to consume educational technologies built in this format.

## Conclusion

The interactive educational material elaborated called “Take care of your heart” was considered valid by the PLHIV. It is also noteworthy that it was an unprecedented study on this topic in Brazil, and that it allowed creating a product consisting in relevant contents, easily accessible and freely available to the population. 

From this, the relevance of the clinical validation process with the target population is highlighted, as the material elaborated must be easy to understand and read by those for whom it is intended, so that its objective can be achieved. 

It is suggested that new studies address the application of this e-book for specific population groups, such as older adults, women, and low-educated and low-income people living with HIV, in order to identify new gaps that may have not yet been addressed.
